# Effects of Storage Time on Glycolysis in Donated Human Blood Units

**DOI:** 10.3390/metabo7020012

**Published:** 2017-03-29

**Authors:** Zhen Qi, John D. Roback, Eberhard O. Voit

**Affiliations:** 1 Department of Biomedical Engineering, Georgia Institute of Technology and Emory University School of Medicine, Atlanta, GA 30332, USA; eberhard.voit@bme.gatech.edu; 2 Center for Transfusion and Cellular Therapy, Department of Pathology and Laboratory, Emory University School of Medicine, Atlanta, GA 30322, USA; jroback@emory.edu; 3 Emory University Hospital, Blood Bank, Atlanta, GA 30322, USA

**Keywords:** blood storage, dynamic model, metabolomics, red blood cells, storage time effect, systems biology

## Abstract

*Background*: Donated blood is typically stored before transfusions. During storage, the metabolism of red blood cells changes, possibly causing storage lesions. The changes are storage time dependent and exhibit donor-specific variations. It is necessary to uncover and characterize the responsible molecular mechanisms accounting for such biochemical changes, qualitatively and quantitatively; *Study Design and Methods*: Based on the integration of metabolic time series data, kinetic models, and a stoichiometric model of the glycolytic pathway, a customized inference method was developed and used to quantify the dynamic changes in glycolytic fluxes during the storage of donated blood units. The method provides a proof of principle for the feasibility of inferences regarding flux characteristics from metabolomics data; *Results*: Several glycolytic reaction steps change substantially during storage time and vary among different fluxes and donors. The quantification of these storage time effects, which are possibly irreversible, allows for predictions of the transfusion outcome of individual blood units; *Conclusion*: The improved mechanistic understanding of blood storage, obtained from this computational study, may aid the identification of blood units that age quickly or more slowly during storage, and may ultimately improve transfusion management in clinics.

## 1. Introduction

About 15 million red blood cell (RBC) units are transfused in the United States every year [1]. It is known that biochemical and biomechanical changes occur during storage and contribute to RBC storage lesion. In addition, recent data suggest that these changes vary among donors and may affect the length of time for which these units can be stored and used [2,3,4]. As a consequence, many blood units donated by healthy donors showed recovery rates as low as 40% of RBCs (red blood cells) after transfusion [2]. The transfusion of these defective RBC units could adversely affect thousands of patients annually and raises the following questions. What are the effects of storage on the metabolism of RBCs from different donors? How are biochemical reactions in RBCs affected during long-term storage? Answering such questions will give us a better mechanistic understanding of storage caused changes of donated blood units and will likely help improve blood storage and transfusion. 

Current FDA (Food and Drug Administration) regulations impose a categorical limit of 42 days for RBCs stored with additive solutions at 4 °C. However, blood units from different donors show drastically different 24-h in vivo survival rates even under the exact same storage conditions [2]. As a case in point, RBCs from 12% of healthy donors, stored in ADSOL (additive solution) medium for 42-days, did not meet the FDA cutoff (75% post-transfusion recovery) [2]. In contrast, some donated units were stored as long as 100 days and still satisfied the FDA standard [5]. Similarly, ATP and hemolysis levels change during storage in ways that not only vary among individuals, but also appear to be heritable [3,4]. What happened in these inferior—or superior—blood units during storage is presently unclear. In other words, personalized storage characteristics have not been taken into account in the clinical practice of transfusion medicine. 

In this study, we investigated the dynamic effects of storage on biochemical reactions in RBCs during 42-day storage with ADSOL additive solution. To monitor the biochemical state of RBCs, metabolites can be measured through mass spectrometry and other technologies at successive time points. The resulting metabolomics data are very informative, but in themselves do not reveal the mechanisms responsible for the observed alterations. In particular, the dynamics of metabolic fluxes, which determine changes in metabolites within RBCs, is ill-understood and needs to be elucidated and quantified. 

To address this issue, we used an approach combining metabolomics time series data, a stoichiometric representation, kinetic models, and customized computational algorithms. Instead of relying on the purification and assessment of individual enzymes, our systems approach integrates metabolomics data, mathematical models, and computational algorithms. It does so in a fundamentally different manner than typical modeling studies, as it is not based on kinetic simulations and direct data fitting. Instead, the computational methods were used to quantify the characteristics of fluxes, as well as the effects of storage on biochemical reactions that are responsible for observed metabolic alterations in stored RBCs. The proposed method is certainly not the final word on storage lesion, but should be seen as a proof of principle for the feasibility of quantitative inferences regarding flux characteristics from metabolomics data. The results from this study are hoped to improve our mechanistic understanding of metabolic alterations during blood storage and may be used to perform in silico tests of new assays for the detection of defective blood units and to predict biochemical outputs of transfusing individual blood units.

## 2. Results

### 2.1. Time Courses of Metabolite Levels. 

Absolute, calibrated metabolite concentrations are shown for the donors of group2 and group2_match in Figure S1 of Supplementary Materials and for donors of group1 in Figure S2. These metabolites have absolute concentrations of different magnitudes. Glucose (GLC), lactate (LAC), and inorganic phosphate (Pi) have concentrations at the order of mM or higher, whereas other concentrations are in the range of 1 to 100 µM. Consistently throughout the different samples, GLC is being used by the cells and therefore shows a monotonic decrease during storage in all donors. The end product LAC exhibits a corresponding monotonic increase. Interestingly, donor X867 shows much higher levels of dihydroxyacetone phosphate (DHAP), and this difference among donors exists in two batches of donations. A mechanistic or molecular explanation of this observation, e.g., changes in another relevant pathway, cannot be given at this point and will require further, targeted research.

### 2.2. Effect of Storage Time

**Figure 1:**
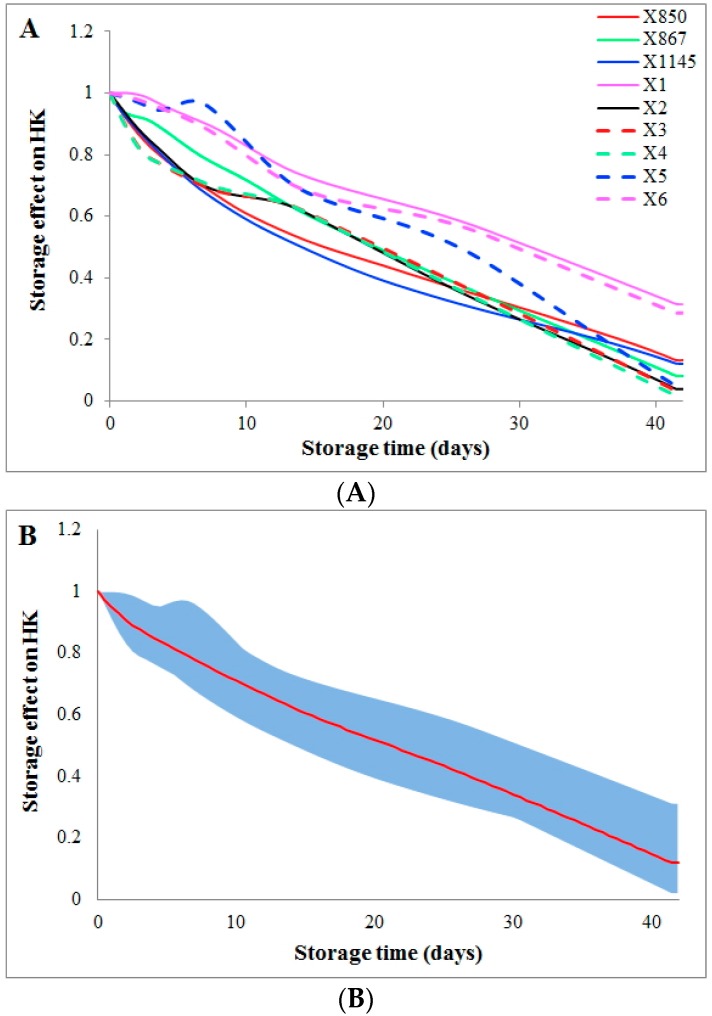
Storage time effects on the HK flux among all donors. Storage time effects on the HK flux are quantified and compared among all donors. Coloring schemes and line styles are defined in the inset legend. The *X*-axis represents storage time (unit: days), while the *Y*-axis shows the effect on the activity of the enzyme HK. (**A**) Two batches of donations from the same donor (group2 and group2_match) are averaged to represent the donor; (**B**) All donations are averaged (the red curve) and the dynamic ranges of variations among donors during storage are shown as the blue area.

The effect of storage time on the flux HK (hexokinase) can be quantified through a comparison between the computed dynamic flux from a kinetic model [6] and the flux directly derived from the metabolomics data. This comparison shows that storage time causes a gradual, almost linear decrease in activity of the enzyme HK from the beginning of storage to the end (Figure 1A). This reduction in HK activity is consistent among donors from two groups (group1 and group2). In addition, the variations in storage time effect among all donors are very similar after the first week of storage, as shown by the blue area (Figure 1B).

The effects of storage time on the flux PK (pyruvate kinase) are similarly determined (Figure S3). Specifically, storage time greatly reduces the activity of PK during the 1st week, after which it reaches a plateau without further decrease until about week four or five. During the 1st week, storage quickly reduces the flux PK by ~80% (Figure S3B). Interestingly, the decreasing trends in HK and PK are quite similar to recent findings by Prudent’s group ([7]).

The same types of analyses were performed for other fluxes within glycolysis, including PFK (phosphofructokinase), ALD (aldolase), and TPI (triose phosphate isomerase) (Figure S4). Each exhibits unique dynamic responses to long-term storage. Storage time does not reduce the flux ALD during the first three weeks, but then quickly slows down this reaction. Donor variations are small, especially during the second half of six week storage. In contrast, donor variations in the effect of storage time on the flux TPI are rather large around the 3rd week of storage.

**Figure 2:**
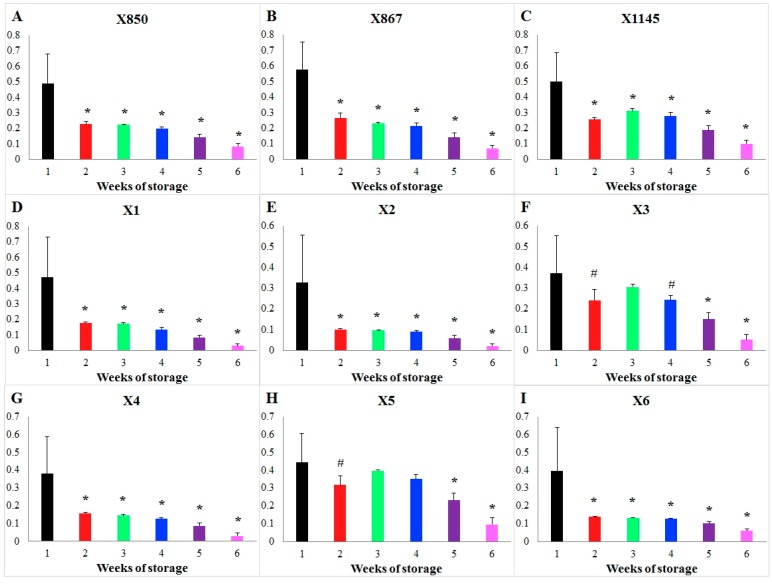
Comparison of the storage time effect on the enzyme PK during different weeks of storage for each donor. Storage time effects on the PK flux are compared week by week for each donor. Bar graphs show the weekly averaged storage effects and standard deviations. The *X*-axis represents storage time (unit: weeks), while the *Y*-axis shows the effect on the activity of the enzyme PK. Each subplot represents the comparison in a donor whose ID is shown in its title, (**A**) donor X850; (**B**) donor X867; (**C**) donor X1145; (**D**) donor X1; (**E**) donor X2; (**F**) donor X3; (**G**) donor X4; (**H**) donor X5; (**I**) donor X6. Significance level: * (*p* < 0.01) for the statistical difference between a corresponding week (2–6) and the 1st week in terms of weekly averaged storage time effects.

Focusing on blood units stored for a short vs. long duration, Figure 2 shows the results of this type of comparison of storage time effects on the flux PK for each individual donor as an example. For each donor, the weekly averaged storage time effects during Weeks 2–6 were compared against that of Week 1 and the statistical significance of the differences was tested. Then, the patterns of significant differences were compared among all donors. Interestingly, storage time changes the flux PK significantly during every week in all donors except for donors X3 and X5. In these two exceptional cases, no significant differences in the effects of storage time were detected when one compares Weeks 2 to 4 against Week 1 (*p* > 0.01). What makes these cases different cannot be explained at the present time. Figure S5 shows a similar comparison for flux HK. In this case, one only finds donor similarity but not specificity.

### 2.3. Influence of Donation Batch

Possible influences of donation batches were quantified for blood donations from the same donors. As an example, Figure S6 exhibits the differences in storage time effect on HK between two batches of donations for each donor. Interestingly, batch differences increase during the first two weeks of storage, but then decrease until the end of the six-week storage. Particularly large batch differences can be seen between the 2nd and 4th weeks of storage. In consideration of different flux magnitudes from individual donors, the batch differences were normalized to the average of two batches from each donor and were shown as relative batch differences in Figure S6. Relative batch differences in the effects of storage time show similar dynamics as the absolute batch differences but with a time shift for each donor. At the later period of storage, one donor constantly showed large relative batch variations, while another donor showed small relative batch differences. One immediate question is whether batch variations are larger than donor differences or vice versa. Such a comparison for HK shows that donor variations are much smaller than batch variations, especially during the 2nd and 4th weeks of storage (Figure S6B). When two donation batches are averaged, the batch variations are removed and the result is a quite similar storage time effect for different donors (please cf. Figure 1).

### 2.4. Comparison of Storage Time Effects among Various Reactions

**Figure 3:**
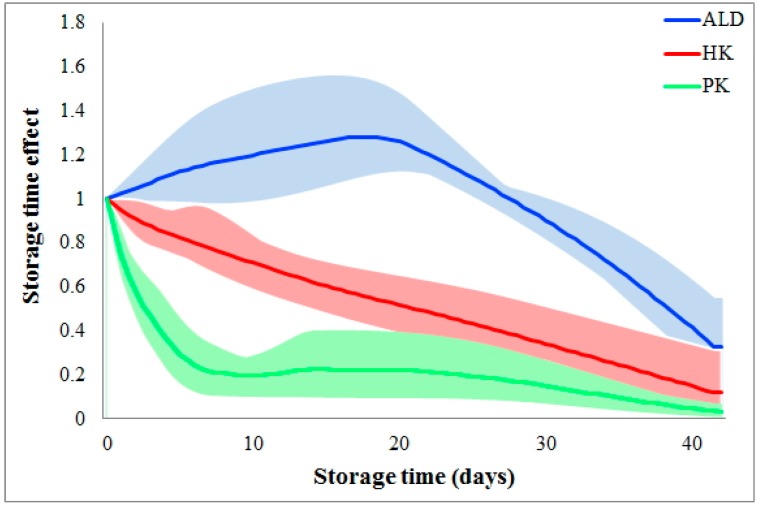
Comparison of storage time effects among fluxes. Storage time effects are compared among fluxes HK, PK, and ALD. Solid color lines represent the averages of storage time effects on each flux among donors, while the colored areas indicate variations. The *X*-axis represents storage time (unit: days), while the *Y*-axis shows storage time effects.

Storage time effects are reaction dependent. Figure 3 shows the different storage time effects among the various glycolytic metabolic steps. As shown, storage time quickly slows the reaction PK during the 1st week and then maintains a small flux during the following five weeks. Differently, the flux HK is almost linearly reduced during the whole six weeks of storage. The third type of storage time effect is shown for the flux ALD, where the storage time does not slow down this flux until Week 4. In fact, ALD initially increases, which could indicate a metabolic shift to other pathways which, however, our analysis does not include. Of note is that the reaction dependency of storage time effects is consistent among donors (please refer to the colored areas in Figure 3). 

### 2.5. Influence of Selecting Particular Kinetic Models

To investigate the influence of the choice of a kinetic model on the quantification of storage time effects on the dynamics of a flux, we compared two kinetic models for the flux PFK from the literature [8,9]. These models assume different reaction mechanisms, with one being based on a unidirectional reaction and the other assuming a reversible reaction. Even so, the results demonstrate consistent storage time effects on the flux PFK for each donor regardless of the model used (Figure S7). For this flux, the storage time effects are very different among donors, but variations from the two models are small, as shown by the grey areas in Figure S7.

### 2.6. Summary of Storage Time Effects

The effects of storage time on various glycolytic reactions in RBCs are summarized in Table 1. Complementing this table, Table S2 shows how the storage time effects themselves can be modulated by other factors. The results clearly demonstrate that storage time affects the different reaction steps within glycolysis in red blood cells quite differently. In addition, the results suggest donor specificity as well as donor similarity. For instance, donor specificity can be seen for two donors (X3 and X5) in comparison with the other seven donors in terms of weekly averaged changes in the effect of storage time on the flux PK. By contrast, there is no such donor specificity for the flux HK. Batch variations among storage effects may actually be larger than donor variations; an example is the flux HK.

## 3. Discussion

The FDA imposes a strict 42-day limit on the storage of RBCs with approved additives at 4 °C. Under these circumstances, donated RBCs are expected to have a 24-h post-transfusion recovery above 75% after 42 days of storage [10]. However, many blood units donated by healthy donors do not meet the FDA standard, and exhibit recovery rates as low as 40% [2], while others satisfy this standard [5]. The reason is that biochemical and biomechanical changes occur during storage and cause storage lesion with individual variations. This storage lesion can be disconcerting, because the usage of deteriorated blood units in transfusion medicine is an important healthcare issue.

The effects of storage on metabolites have been studied before. For instance, Roback and coworkers characterized metabolites in various pathways from stored blood units sampled from multiple donors [11]. Other investigators showed metabolic variability in RBCs from different donors [3,4]. Bordbar, Paglia, and others have used metabolomics and statistical analysis to correlate storage time with metabolite levels and to find biomarkers with phasic correlations [12,13]. Others have monitored the time-course of metabolites during cold storage of red blood cell concentrates [14,15,16,17,18,19], investigated the effects of genetically distinct inbred strains of mice on RBC storage and transfusion [20], quantified bioactive lipid accumulation in leukoreduced RBC units with large donor-to-donor variations [21], or correlated peroxidation of lipids with post-transfusion circulation of stored murine RBCs [22]. Our analysis here is complex as it targets not the concentrations of metabolites, but alterations in the biochemical processes that lead to the changed metabolite profiles.

Thus, the challenge of our proof-of-principle analysis was to infer and characterize, qualitatively and quantitatively, some of the mechanisms responsible for these changes. Metabolomics data demonstrate the dynamics of metabolites in RBCs during long-term storage, but do not reveal the underlying mechanisms themselves. In this study, we address this challenge by integrating metabolomics data, a stoichiometric model, kinetic rate functions, and customized computational algorithms. The results suggest that storage time affects many reaction steps of glycolysis in RBCs and that these changes are reaction specific. Table 1 lists important, qualitatively distinct types of such storage time effects. Interestingly, others used mass spectrometry to dynamically monitor metabolic species during cold storage of erythrocyte concentrates and found very different dynamic levels of glycolytic metabolites, which may support reaction dependent effects of storage time [14]. 

Of course, many explanations for storage lesions have been proposed. Based on a murine model of RBC storage, Zimring and colleagues found substantial strain-specific differences in post-transfusion RBC recovery [20]. Delobel et al. pointed to carbonylation of cytoplasmic and membrane proteins along storage, which indicates protein oxidative lesions and could be one of many possible mechanistic explanations for storage lesions [23]. Reisz and coworkers discovered oxidation of GAPDH without exogenous excess pro-oxidant compounds in stored blood units [24]. Nishino and colleagues used a computational analysis of metabolome to study the influence of adenine and guanosine on ATP and 2,3-BPG [25]. Bordbar and colleagues used statistical methods to analyze metabolite levels during blood storage [12], and Paglia and coworkers used classifier and receiver operating characteristic curve analysis to distinguish three metabolic states [13].

The overall conclusion from this study can likely be extrapolated to fluxes in other metabolic pathways, but this expectation is yet to be confirmed. Interestingly, some donors showed distinct patterns in their flux dynamics in response to storage, which may be a root cause for individual variations in blood transfusion outcomes. For example, two donors X3 and X5 are distinct from others by showing no difference in storage time effect on the flux PK between Week 1 versus Weeks 2–4 of storage, while all other donors do show changes in storage effects in a week-by-week comparison. It should be emphasized that this study used metabolomics data from a limited number of donated units and thus suggested individual variations should be further investigated with a much larger cohort. Because of missing measurements of some metabolites, we were not able to study the effects of storage time on some other processes within RBC metabolism, such as G6PDH. With regards to this process, Tzounakas and coworkers performed interesting studies in regards to the relationship between G6PDH and donor variations in transfusion medicine [26,27]. Similarly interesting, Peters and colleagues measured the activity of G6PDH in individual RBCs and found that mean fluorescent intensity per RBC after 3 days of storage was 27.8 ± 8.8 and gradually decreased significantly to 18.0 ± 8.3 after 42 days [28].

While preliminary, this study led to other interesting conclusions. For example, it shows that the influence of the donation batch can be larger than donor variations within each batch. However, one needs to keep in mind that the metabolomics data used in this study are from a limited number of donors, sampled at a relatively small number of time points, and monitor only a subset of metabolites. Due to technological limitation, the dataset used in this study contains relative values which were converted into absolute concentrations with an assumption of a linear correlation. This can be improved by using external calibration with reference standards [12]. Gevi et al. used fold changes in metabolic species between storage and day 0 controls [14]. While these measurements can certainly be improved in time resolution, quantification, comprehensiveness, accuracy, and from more donors, the study provides a glimpse at the potential that such data, integrated with different mathematical models and computational algorithms, have for a better understanding of biochemical mechanisms associated with observed metabolic changes during blood storage.

*Limitations of the data and their analysis.* It is necessary to emphasize the limitations of the dataset and the analyses used in this study. First, due to technological limitations, the dataset used in this study, which has been published [11], consists of relative concentrations. Although relative measurements are often used in metabolomics, they do pose a challenge to the development of precise models (reviewed in [29]), to which absolute measurements can provide help. Our approach will have no problem with the analysis of absolutely quantified metabolomics data, which will more likely become available for computational analyses. Second, our approach requires kinetic formulae and their parameter values in order to quantify the storage effects on the corresponding enzymatic reactions. Through over a century of efforts by biochemists, ample kinetic information has been accumulated and can be found on databases like BRENDA (http://www.brenda-enzymes.org/). Very often, there are multiple formulae available for the same reaction, and the choice of a kinetic formula could possibly alter the quantification of storage effects. In the limited exploration of this study, we did show that different kinetic formulae can result in consistent quantification of storage time effects on the flux PFK for each donor regardless of the model used. Third, the metabolomics data used in this study are from a limited number of donors, sampled at a relatively small number of time points, and monitor only a subset of metabolites. Thus, more data need to be analyzed by our approach before the results and predictions in this study can be generalized.

## 4. Materials and Methods

### 4.1. Metabolomics Data

Research donors were screened by health history questionnaire and vital signs and provided consent to donate whole blood. Twelve units were donated by nine volunteers (please refer to Table S1 for demography), with one unit from each of six volunteers (group1, donors: X1, X2, X3, X4, X5, X6) and two units with a gap of several months from each of three additional volunteers (group2 and group2_match, donors: X850, X867, X1145). After leukoreduction and removal of platelet-rich plasma, the residual RBC pellet was mixed with ADSOL additive solution, and the packed RBC unit was stored at 2–6 °C for up to 42 days. At selected time points, RBC bags were gently but thoroughly mixed, and 1 mL samples were aseptically removed, added to labeled cryovials, snap frozen on liquid nitrogen, and stored at −80 °C. All samples were then analyzed with gas chromatography/mass spectrometry and liquid chromatography/tandem mass spectrometry. Specific compounds were identified by comparison to library entries of purified standards or recurrent unknown entities. More technical details can be found elsewhere [11].

### 4.2. Factors Influencing Metabolic Flux Dynamics during Storage

This study mainly targets the quantification of the effects of storage time (during six weeks of refrigerated storage) on metabolic fluxes in red blood cells under the specified storage condition. We also analyzed and quantified the influence of donation batch (same group of volunteers donated two times with several months between donations), differences in effects for different reactions, and the selection of kinetic models for the dynamic quantification of storage time effects (Section 3 in Supplementary Materials).

### 4.3. Pathways of Importance

**Figure 4:**
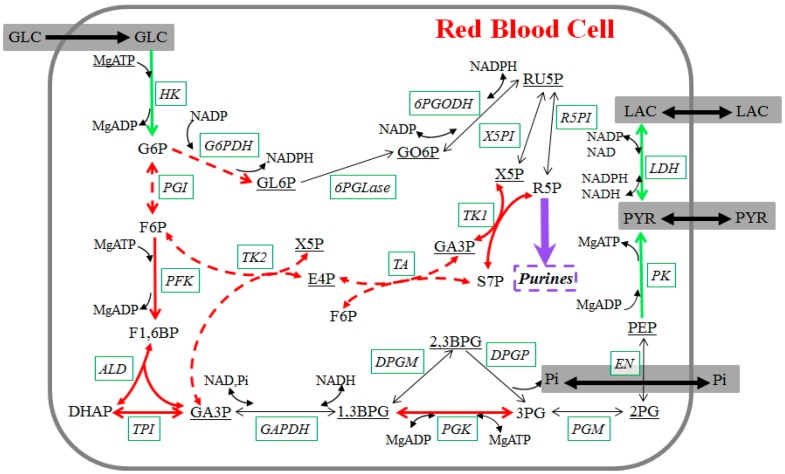
Glycolysis in stored human red blood cells. Measured metabolites are shown in straight font; underlined metabolites were not measured. Enzymes are shown in italics with solid boxes. Reactions are represented with arrows. Green arrows are fluxes whose dynamics during storage can be directly derived from the metabolomics data; dashed red arrows indicate fluxes where additional assumptions were made, and red arrows show reactions whose dynamics can be derived with the inclusion of these assumptions. The purple arrow represents the main flux toward purine metabolism. Black arrows are fluxes whose dynamics cannot be derived. Regulatory signals are omitted for a clearer visualization. Metabolites, enzymes, and their abbreviations are: glucose (GLC), glucose 6-phosphate (G6P), fructose 6-phosphate (F6P), fructose 1,6-bisphosphate (F1,6BP), dihydroxyacetone phosphate (DHAP), glyceraldehyde 3-phosphate (GA3P), 1,3-bisphosphoglycerate (1,3BPG), 2,3-bisphosphoglycerate (2,3BPG), 3-phosphoglycerate (3PG), 2-phosphoglycerate (2PG), phosphoenolpyruvate (PEP), pyruvate (PYR), lactate (LAC), gluconolactone 6-phosphate (GL6P), gluconate 6-phosphate (GO6P), ribulose 5-phosphate (RU5P), xylulose 5-phosphate (X5P), ribose 5-phosphate (R5P), sedoheptulose 7-phosphate (S7P), erythrose 4-phosphate (E4P), adenosine monophosphate (AMP), adenosine diphosphate (ADP), adenosine triphosphate (ATP), nicotinamide adenine dinucleotide phosphate (NADP), nicotinamide adenine dinucleotide phosphate (NADPH), nicotinamide adenine dinucleotide (NAD), nicotinamide adenine dinucleotide (NADH), inorganic phosphate (Pi), magnesium (Mg), hexokinase (HK), phosphoglucoisomerase (PGI), phosphofructokinase (PFK), aldolase (ALD), triose phosphate isomerase (TPI), glyceraldehyde phosphate dehydrogenase (GAPDH), phosphoglycerate kinase (PGK), 2,3-bisphosphoglycerate-dependent phosphoglycerate mutase (DPGM), 2,3-diphosphoglycerate phosphatase (DPGP), phosphoglyceromutase (PGM), enolase (EN), pyruvate kinase (PK), lactate dehydrogenase (LDH), glucose 6-phosphate dehydrogenase (G6PDH), 6-Phosphogluconolactonase (6PGLase), 6-Phosphogluconate dehydrogenase (6PGODH), xylulose 5-phosphate isomerase (X5PI), ribose 5-phosphate isomerase (R5PI), transaldolase (TA), transketolase1 (TK1), transketolase2 (TK2).

We focus primarily on changes in glycolytic activity during 42-day blood storage (Figure 4), but also account for contributions from the pentose phosphate pathway (PPP). Mature RBCs synthesize ATP exclusively through glycolysis, because they lack mitochondria and the cytochrome system necessary to perform oxidative phosphorylation. Within glycolysis, the molecule 2,3-bisphosphoglycerate (2,3BPG) is an important intermediate metabolite, because it can regulate the carriage and release of oxygen by hemoglobin. Furthermore, NADPH, generated via PPP, converts the oxidized form of glutathione to its reduced form, which is necessary to maintain the structure of RBCs and to keep hemoglobin in the ferrous state.

### 4.4. Calibration of Metabolomics Data

The levels of metabolites in RBCs stored with ADSOL (additive solution) were measured at several time points during storage; technical details are described elsewhere [11]. A brief description of the data collection methods and the subsequent mass spectrometry analysis can be found in the Section 1 in Supplementary Material. The data were obtained from twelve units donated by nine volunteers (Table S1). Among these, six volunteers each donated one unit (group1, donors: X1, X2, X3, X4, X5, X6), while three additional volunteers donated two units with several months between the two batches of donations (group2 and group2_match, donors: X850, X867, X1145, using the nomenclature in [11]). 

The dataset contains relative values that need to be converted into absolute concentrations for our investigation. This conversion assumes that absolute concentrations exhibit a linear correlation with mass spectrometry signals, so that the higher the mass spectrometry signal is, the higher an absolute concentration will be. As a baseline, the average concentration of a metabolite among donors at day 0 is assumed to be the same as that for the general population, which was surveyed from the literature. The conversion of concentrations is separately performed for each metabolite and takes into consideration the fact that mass spectrometry signals depend on biophysical and biochemical characteristics of specific metabolites. Of course, it would have been preferable to be able to use absolute metabolite levels in our model. However, such data were not available, forcing us to rely on relative concentrations. Nonetheless, it was pointed out in a recent, comprehensive review of the state of the art in metabolic systems modeling that similar approaches are commonly based on relative measurements [29]. Specifically, the authors state: “…most studies, wherein large-scale targeted metabolomics were performed, report metabolic abundances in relative concentrations, or peak intensities, rather than in absolute concentrations”. Thus, while not ideal, we pursued our investigation with relative quantities.

After the conversion to absolute concentrations, the data were calibrated according to the signal intensity of carbon atoms in consideration of 5–6% instrument variability and 13–18% total process variability [11]. This calibration allowed a more accurate comparison of samples collected at different time points from multiple donors. Since stored RBCs constitute an isolated system, we can expect that carbon atoms do not disappear but redistribute among metabolites during storage. Thus, the sum of all metabolite masses and the number of carbon atoms from a measured metabolite at any time point can be compared with the corresponding quantity at day 0, which allows us to normalize metabolite concentrations using the following formula:

(1) $${\left[normalized\;metabolite\right]}_{t}={\left[metabolite\right]}_{t}\times \frac{{\left[carbon\;atom\right]}_{0}}{{\left[carbon\;atom\right]}_{t}}$$

Here, the subscript 0 indicates day 0 and [*normalized metabolite*]*_t_* represents the normalized concentration of a metabolite at time *t* using concentrations of carbon atoms at days 0 and *t*: [*carbon atom*]_0_ and [*carbon atom*]*_t_*.

**Figure 5:**
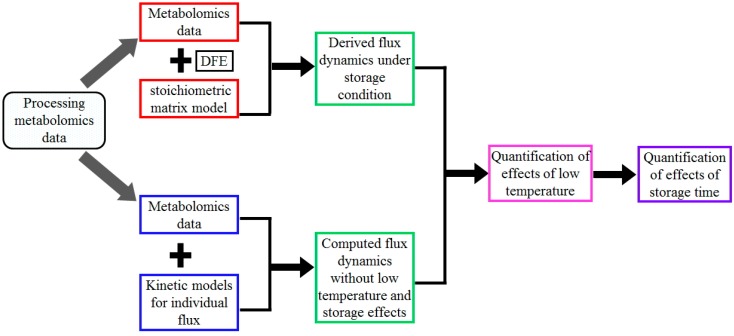
Flow chart for the quantification of storage effects using the QDEC method. The chart shows the steps involved from data processing to storage effect quantification. The step “Processing metabolomics data” comprises the conversion into absolute concentrations, followed by data calibration using the signal intensity of carbon atoms. Then two different approaches were applied: (1) Derive fluxes under storage conditions from metabolomics data at discrete time points using the method of dynamic flux estimation (DFE) and the stoichiometric modeling approach (Steps “Metabolomics data” + “DFE” + “stoichiometric model” + “Derived flux dynamics under storage conditions”). Among them, the stoichiometric model characterizes the relationships between the dynamic changes in metabolite concentrations and glycolytic fluxes, while DFE uses the stoichiometric model and computed derivatives of metabolite concentrations at discrete time points to infer some fluxes using the inverse of the stoichiometric matrix (or its pseudo-inverse if the matrix is under-determined); (2) Compute fluxes under normal physiological conditions at discrete time points using kinetic formalisms for each individual reaction together with measured metabolic profiles (Steps “Metabolomics data” + “Kinetic models for individual flux” + “Computed flux dynamics excluding low temperature and storage effects”. With the same metabolic profiles, the two sets of fluxes differ in the effects of normal physiology vs. storage at discrete time points. Their comparison at day 0 (Step “Quantification of effects of low temperature”) only shows low temperature effects, while their comparisons after the removal of low temperature effects at other time points (Step “Quantification of effects of storage time”) show the storage effects.

As a schematic illustration, Figure 5 shows the flow chart for the analysis of metabolomics data and the quantification of storage effects using the proposed “quantification of dynamic enzymatic change” (QDEC) method, whose details are discussed in the following.

### 4.5. Strategy for the Quantification of Storage Time Effects on Flux Dynamics in Stored Blood

Our task is to study how storage mechanistically affects RBC metabolism. We pursue this task by quantifying alterations in the dynamics of fluxes, which are due to changes in enzymatic activities during storage. The QDEC method, which we propose for this purpose, proceeds in the following manner. First, we infer, directly from the calibrated metabolomics data, the dynamics of fluxes under the given storage regimen. Second, we use published information to compute the corresponding flux dynamics under normal physiological conditions. Third, we compare and quantify the differences between these two sets of flux values, one metabolite measurement at a time, and conclude that they are due to the effects of low temperature and storage time. In addition, we assess the influence of the donation batch, differences in effects for different reactions, and the selection of particular kinetic models on the dynamic quantification of storage time effects. One should note that this strategy differs fundamentally from traditional simulation and parameter estimation studies. This strategy directly accounts for pertinent regulation, and ultimately reveals different storage effects at each time point.

### 4.6. Inference of Flux Dynamics from Metabolomics Data under Storage Conditions

A stoichiometric model was developed for the glycolytic system and PPP. It characterizes the relationships between dynamic changes in metabolite concentrations and glycolytic fluxes (for details, see Section 2 in Supplementary Material). Because metabolomics data are available, the method of dynamic flux estimation (DFE) allows the direct quantitative derivation of many of the glycolytic fluxes (see [30,31] for technical details regarding DFE). In order to infer the remaining fluxes, we imposed reasonable biological constraints, such as the observation that the flux split ratio between glycolysis and PPP at glucose 6-phosphate (G6P) tends to be approximately 90% vs. 10%. The generic result of this step is a numerical representation of each flux, either as a function of time or as a function of the kinetic variables affecting it. 

### 4.7. Quantification of Fluxes under Normal Physiological Conditions and Comparison with Storage Conditions

The previous section described the computational derivation of fluxes from the metabolomics data during storage. Now, we estimate the corresponding fluxes under normal physiological conditions. Then, it becomes possible to compare the two sets of fluxes and quantify the effects of low temperature and storage time on enzyme activities. This approach uses the new QDEC method instead of a typical simulation approach of a kinetic model because the latter is not feasible in this situation. A kinetic formalism can capture a biochemical process mathematically and describe its dynamics. All kinetic formalisms together form a kinetic model, which has a determined symbolic model, parameter values, and an initial condition. Such a kinetic model can then be simulated and used to study the dynamics of the system. However, the storage of red blood cells can result in dynamic changes in enzymatic activities, which are also evident in the present study; thus the traditional simulation approach is not applicable without explicitly knowing the dynamics of changing parameters. To quantify the changing enzymatic activities, the QDEC method, which does not use the typical simulation approach, is proposed in this study. Instead, this new method uses measured metabolic profiles and kinetic formalisms from the literature to compute reaction rates of each individual reaction at discrete time points under normal physiological conditions and to compare them with the inferred fluxes under the storage condition. It does not simulate the dynamics of metabolic profiles, which have been represented by the measurements, through a kinetic model and integration of ODEs. Therefore, it does not need initial concentrations or ODE integrations to quantify the dynamic changes in enzymatic activities. Because of this feature, the influence of variations of initial values on the results and conclusions is greatly eliminated.

Several publications have proposed fully parameterized kinetic models of glycolytic enzymes in RBCs under normal physiological conditions. We surveyed the literature and selected kinetic formulations that include known regulatory signals [32,33,34,35,36,37] (Section 2 in Supplementary Material). It is reasonable to expect that these regulatory signals are also present during refrigerated storage. Entering the reported, normal enzyme activities, measured concentrations from the metabolomics data, levels of regulators, and parameter values into the published kinetic formulations generates a set of computed fluxes under normal physiological conditions. Their comparison with actually inferred flux values (previous section) quantifies the alteration in enzyme activity. This analysis is performed at discrete time points throughout the entire storage time period of 42 days. 

### 4.8. Effect of Storage Time on Flux Dynamics

The thus obtained two sets of flux dynamics, one under storage conditions inferred from the metabolomics data and the other under normal physiological conditions, computed with kinetic models from the literature, differ in the effects of low temperature as well as storage time. The comparison between these two sets of flux dynamics allows the quantification of effects of these two factors, which we assume to affect enzymatic activities in a multiplicative fashion. Furthermore, samples of stored RBCs at day 0 were considered as being affected only by low temperature, but not by storage time. As a consequence, the Day-0 samples show only the effect of low temperature and this effect can be quantified and kept constant for the rest of storage. Finally, because the blood units had been stored at about 4 °C, the temperature was considered constant during storage, and its effect on a particular reaction was therefore expected to remain the same. With this deconvolution, the specific effect of storage time on each flux can be quantified.

### 4.9. Influence of Other Factors on the Dynamic Quantification of Storage Time Effects

Other factors can influence the dynamic quantification of storage time effects and the consequent conclusions. These factors include: different donation batches and the selection of kinetic models for the computation of the expected flux dynamics under normal physiological conditions. In addition, we assess variations among reactions in storage time effects. For the quantification of batch effects, we compared two batches of donations by the same donors with an interval time period of several months in between. Both the absolute differences in storage time effects between the two batches (group2_Match vs. group2) for each donor and their relative values to the average of the two batches were computed. For example, consider donor X850 and suppose the effect of storage time on the flux HK is batch dependent and is *Y*(*t*, *batch*). Then, the absolute differences between two batches are *Y*(*t*, *batch1*) − *Y*(*t*, *batch2*), while the relative differences are (*Y*(*t*, *batch1*) − *Y*(*t*, *batch2*))/*average*(*Y*(*t*, *batch1*), *Y*(*t*, *batch2*)) × 100%.

*Ethics Approval and Consent to Participate:* Research donors were screened by health history questionnaire and vital signs and provided consent to donate whole blood. The metabolomics data from these donations consist of measurements of serially sampled units of leukoreduced ADSOL red blood cells (RBCs) over 42 days of refrigerated storage. The Institutional Review Board at Emory University approved all protocols.

*Availability of Data and Material:* The datasets used and/or analyzed during the current study as well as relevant technical details can be found in a published paper [11].

## 5. Conclusions

To quantify the effects of storage time, we developed a novel method that exceeds traditional parameter estimation methods, which are not applicable here because the parameters for storage effects are not constant but change as functions of time. The effects of low temperature and storage time were explicitly distinguished because factors such as temperature are reversible and can be restored during transfusion, while enzymatic degradation due to storage time is likely irreversible. 

While these results are intriguing, they are preliminary, and this study is clearly not the last word on blood storage. However, this study offers a glimpse into the potential of combining sophisticated metabolomics experiments with custom-tailored computational analyses.

Our investigation detected distinct patterns of flux dynamics in stored RBCs from some donors, and these may be the cause of donor specific storage lesion and different transfusion outcomes. These potential relationships will require further experimental investigation. The donor specificity suggests the consideration of personalized transfusion diagnostics and usage, which would replace statistical averages. To accomplish such differentiation, experimental and computational methods as described here would have to identify biomarkers, which could then be used for tests of individual blood donations that had been stored for several weeks.

Using kinetic models, we were furthermore able to disentangle and quantify reversible factors, such low temperature (4 °C), regulation by metabolites, pH, and irreversible processes such as protein degradation. Other studies have already characterized the changes in proteins during blood storage, and data from these studies could be very useful for computational simulations and comparisons with our results [38,39,40,41]. Accounting for these factors is the basis for simulating rejuvenation and transfusion processes and for predictions of the metabolic dynamics of an individual blood unit. For instance, during rejuvenation, ATP and 2,3BPG can be restored to approximately normal levels, and temperature is elevated back to the normal body temperature [42,43]. These actions could be put into kinetic models where ATP and 2,3BPG are variables and their levels can be directly changed. Interestingly, D’Alessandro et al. used isotope labeling and metabolic tracer experiments and found that mature RBCs can metabolize supplied substrates [17]. The contribution of temperature is characterized by a parameter and quantified as described before. Thus, the effects of rejuvenation on fluxes and metabolites can be simulated and evaluated. Transfusion can be similarly simulated, when changes in pH and temperature are taken into account. These factors are characterized by specific parameters, whose quantification allows for the simulation of biochemical dynamics after transfusion, especially of the in vivo dynamics within 24-h. One might add that methods similar to those presented here could also be applied to anaerobic storage [18].

A better mechanistic understanding of the metabolism of donated blood units during storage is important for the improvement of transfusion strategies and would certainly benefit many of the 15 million transfusions performed every year. In the future, analyses like those shown here may help clinicians to obtain a fast, useful evaluation of metabolic fluxes before a specific blood unit is transfused that had been stored for several weeks. It could also potentially aid the identification of blood units that would allow for longer storage without compromising the FDA standard.

## Figures and Tables

**Table 1:** Effects of storage time on glycolytic reactions in stored RBCs.

Effect on Specific Reactions	Example
Storage quickly reduces the speed of a reaction during the 1st week and then retains a small flux during the following 5 weeks.	PK
The flux magnitude linearly decreases during the entire 6 weeks of storage.	HK
Storage does not slow down a flux until after 3 weeks.	ALD
Except for the two donors (X3 and X5), there are significant differences in the storage effect between the 1st, 2nd, 3rd, and 4th week.	PK
In all donors, storage significantly changes a flux every week in comparison with the 1st week.	HK

## Supplementary Materials

The following are available online at www.mdpi.com/2218-1989/7/2/12/s1, Figure S1: Dynamics of metabolite levels for donors in group2 and group2_match, Figure S2: Dynamics of metabolite levels for donors in group1, Figure S3: Storage effect on the PK flux among all donors. Storage time effects on the PK flux are quantified and compared among all donors, Figure S4: Storage effect on fluxes PFK, ALD, and TPI. Storage time effects on fluxes PFK, ALD, and TPI are quantified and compared among all donors, Figure S5: Comparison of storage time effect on the HK flux during different weeks of storage for each donor. Storage time effects on the HK flux are compared week by week for each donor, Figure S6: Influence of donation batches on storage time effect on the flux HK, Figure S7: Influence of different kinetic models. Two available kinetic models for the flux PFK were used to quantify storage time effect. Solid lines represent results from one model, while dashed lines are from the other model, Table S1: Demographics of the donors, Table S2: Influence of secondary factors on storage time effects.

## Abbreviations

1,3-bisphosphoglycerate (1,3BPG), 2,3-bisphosphoglycerate (2,3BPG), 2,3-bisphosphoglycerate-dependent phosphoglycerate mutase (DPGM), 2,3-diphosphoglycerate phosphatase (DPGP), 2-phosphoglycerate (2PG), 3-phosphoglycerate (3PG), 6-Phosphogluconate dehydrogenase (6PGODH), 6-Phosphogluconolactonase (6PGLase), adenosine diphosphate (ADP), adenosine monophosphate (AMP), adenosine triphosphate (ATP), aldolase (ALD), dihydroxyacetone phosphate (DHAP), dynamic flux estimation (DFE), enolase (EN), erythrose 4-phosphate (E4P), fructose 1,6-bisphosphate (F1,6BP), fructose 6-phosphate (F6P), gluconate 6-phosphate (GO6P), gluconolactone 6-phosphate (GL6P), glucose (GLC), glucose 6-phosphate (G6P), glucose 6-phosphate dehydrogenase (G6PDH), glyceraldehyde 3-phosphate (GA3P), glyceraldehyde phosphate dehydrogenase (GAPDH), hexokinase (HK), inorganic phosphate (Pi), lactate (LAC), lactate dehydrogenase (LDH), magnesium (Mg), nicotinamide adenine dinucleotide (NAD), nicotinamide adenine dinucleotide (NADH), nicotinamide adenine dinucleotide phosphate (NADP), nicotinamide adenine dinucleotide phosphate (NADPH), pentose phosphate pathway (PPP), phosphoenolpyruvate (PEP), phosphofructokinase (PFK), phosphoglucoisomerase (PGI), phosphoglycerate kinase (PGK), phosphoglyceromutase (PGM), pyruvate (PYR), pyruvate kinase (PK), quantification of dynamic enzymatic change (QDEC) , red blood cell (RBC), ribose 5-phosphate (R5P), ribose 5-phosphate isomerase (R5PI), ribulose 5-phosphate (RU5P), sedoheptulose 7-phosphate (S7P), transaldolase (TA), transketolase1 (TK1), transketolase2 (TK2)., triose phosphate isomerase (TPI), xylulose 5-phosphate (X5P), xylulose 5-phosphate isomerase (X5PI).
