# Supplementary material for: Effects of Storage Time on Glycolysis in Donated Human Blood Units
Source: Metabolites. 2017 Mar 29;7(2):12. doi: 10.3390/metabo7020012 (PMC5487983; doi:10.3390/metabo7020012)
Supplement: Supplementary file 1 [file metabolites-07-00012-s001.pdf]

Supplementary Materials: Effects of Storage Time on Glycolysis in Donated Human Blood Units

Zhen Qi, John D. Roback, Eberhard O. Voit

1. Data collection and mass spectrometry analysis (adapted from [1]).

Research donors were screened by health history questionnaire and vital signs and provided consent to donate whole blood. The metabolomics data from these donations consist of measurements of serially sampled units of leukoreduced ADSOL red blood cells (RBCs) over 42 days of refrigerated storage.

Specifically, the data came from twelve units donated by nine volunteers (please refer to Table S1 for demography). Among these, six volunteers each donated one unit (group1, donors: X1, X2, X3, X4, X5, X6); three additional volunteers donated two units with several months between donations (group2 and group2_match, donors: X850, X867, X1145). After leukoreduction and removal of platelet-rich plasma, the residual RBC pellet was mixed with ADSOL additive solution, and the packed RBC unit was stored at 2–6°C for up to 42 days. At selected time points, RBC bags were gently but thoroughly mixed, and 1 mL samples were aseptically removed, added to labeled cryovials, snap frozen on liquid nitrogen, and stored at −80°C. Samples from each study were stored until all time points were collected and then analyzed with gas chromatography/mass spectrometry and liquid chromatography/tandem mass spectrometry. For the former analysis, samples were dried, derivatized using bistrimethyl-silyl-triflouroacetamide, and run on a Thermo-Finnigan Trace DSQ fast-scanning single-quadrupole mass spectrometer (Waltham, MA) using electron impact ionization. The LC/MS/MS used a Waters ACQUITY UPLC (Milford, MA) and a Thermo-Finnigan LTQ mass spectrometer, consisting of an electrospray ionization source and linear ion-trap mass analyzer. Specific compounds were identified by comparison to library entries of purified standards or recurrent unknown entities. The peak areas for each named metabolite were log-transformed and normalized to Bradford protein content.

The ADSOL medium contained adenine, dextrose, and other ingredients. The possible direct regulations of reaction kinetics by these ingredients were already accounted for by the kinetic formalisms we used. The indirect effects of these molecules, by means of affecting secondary metabolites, are accounted for by the metabolomics data and their usage in the kinetic models and the stoichiometric model.

The Institutional Review Board at Emory University approved all protocols.

2. Stoichiometric model.

A stoichiometric model was developed to quantify the connections between dynamic changes in metabolite concentrations and glycolytic fluxes at each time point, namely:

$$\frac{d[metabolites]}{dt} = [stoichiometric\ matrix] \times [fluxes]$$

Here, the expression [metabolites] represents a column vector containing metabolite concentrations at a time point, while [fluxes] is a column vector containing flux values at the same time point.

As an illustration, the set-up of a stoichiometric model using metabolomics data for the donor in group2_match is shown here:

The [metabolites] column vector contains: [GLC; G6P; F6P; F16BP; DHAP; GA3P; 13BPG; 23BPG; 3PG; 2PG; PEP; PYR; LAC; MgADP; MgATP; Pi; GL6P; GO6P; RU5P; X5P; R5P; S7P; E4P]. Please refer to the legend of Figure 4 for abbreviations.

The [fluxes] column vector contains: [V_13BPG_23BPG_DPGM; V_13BPG_3PG_PGK;

V_23BPG_3PG_DPGP; V_2PG_PEP_EN; V_3PG_2PG_PGM; V_DHAP_GA3P_TPI; V_F16BP_GA3P_ALD; V_F6P_F16BP_PFK; V_G6P_F6P_PGI; V_G6P_GL6P_G6PDH; V_GA3PS7P_E4PF6P_TA; V_GA3P_13BPG_GAPDH; V_GL6P_GO6P_6PGLase; V_GLC_G6P_HK; V_GO6P_RU5P_6PGODH; V_PEP_PYR_PK; V_PYR_LAC_LDH; V_RU5P_R5P_R5PI; V_RU5P_X5P_X5PI; V_X5PE4P_F6PGA3P_TK2; V_X5PR5P_GA3PS7P_TK1]. The format of the flux names starts with the capital letter V, then substrate, product, and the catalyzing enzyme. These four parts within a flux name are separated by an underline symbol. For abbreviations of enzymes, please refer to the legend of Figure 4.

The [stoichiometric matrix] is a 23×21 array:

$$
\begin{bmatrix}
0 & 0 & 0 & 0 & 0 & 0 & 0 & 0 & 0 & 0 & 0 & 0 & 0 & -1 & 0 & 0 & 0 & 0 & 0 & 0 & 0 \\
0 & 0 & 0 & 0 & 0 & 0 & 0 & 0 & -1 & -1 & 0 & 0 & 0 & 1 & 0 & 0 & 0 & 0 & 0 & 0 & 0 \\
0 & 0 & 0 & 0 & 0 & 0 & 0 & -1 & 1 & 0 & 1 & 0 & 0 & 0 & 0 & 0 & 0 & 0 & 0 & 1 & 0 \\
0 & 0 & 0 & 0 & 0 & 0 & -1 & 1 & 0 & 0 & 0 & 0 & 0 & 0 & 0 & 0 & 0 & 0 & 0 & 0 & 0 \\
0 & 0 & 0 & 0 & 0 & -1 & 1 & 0 & 0 & 0 & 0 & 0 & 0 & 0 & 0 & 0 & 0 & 0 & 0 & 0 & 0 \\
0 & 0 & 0 & 0 & 0 & 1 & 1 & 0 & 0 & 0 & -1 & -1 & 0 & 0 & 0 & 0 & 0 & 0 & 0 & 1 & 1 \\
-1 & -1 & 0 & 0 & 0 & 0 & 0 & 0 & 0 & 0 & 0 & 1 & 0 & 0 & 0 & 0 & 0 & 0 & 0 & 0 & 0 \\
1 & 0 & -1 & 0 & 0 & 0 & 0 & 0 & 0 & 0 & 0 & 0 & 0 & 0 & 0 & 0 & 0 & 0 & 0 & 0 & 0 \\
0 & 1 & 1 & 0 & -1 & 0 & 0 & 0 & 0 & 0 & 0 & 0 & 0 & 0 & 0 & 0 & 0 & 0 & 0 & 0 & 0 \\
0 & 0 & 0 & -1 & 1 & 0 & 0 & 0 & 0 & 0 & 0 & 0 & 0 & 0 & 0 & 0 & 0 & 0 & 0 & 0 & 0 \\
0 & 0 & 0 & 1 & 0 & 0 & 0 & 0 & 0 & 0 & 0 & 0 & 0 & 0 & 0 & -1 & 0 & 0 & 0 & 0 & 0 \\
0 & 0 & 0 & 0 & 0 & 0 & 0 & 0 & 0 & 0 & 0 & 0 & 0 & 0 & 0 & 1 & -1 & 0 & 0 & 0 & 0 \\
0 & 0 & 0 & 0 & 0 & 0 & 0 & 0 & 0 & 0 & 0 & 0 & 0 & 0 & 0 & 0 & 1 & 0 & 0 & 0 & 0 \\
0 & -1 & 0 & 0 & 0 & 0 & 0 & 1 & 0 & 0 & 0 & 0 & 0 & 1 & 0 & -1 & 0 & 0 & 0 & 0 & 0 \\
0 & 1 & 0 & 0 & 0 & 0 & 0 & -1 & 0 & 0 & 0 & 0 & 0 & -1 & 0 & 1 & 0 & 0 & 0 & 0 & 0 \\
0 & 0 & 1 & 0 & 0 & 0 & 0 & 0 & 0 & 0 & 0 & -1 & 0 & 0 & 0 & 0 & 0 & 0 & 0 & 0 & 0 \\
0 & 0 & 0 & 0 & 0 & 0 & 0 & 0 & 0 & 1 & 0 & 0 & -1 & 0 & 0 & 0 & 0 & 0 & 0 & 0 & 0 \\
0 & 0 & 0 & 0 & 0 & 0 & 0 & 0 & 0 & 0 & 0 & 0 & 1 & 0 & -1 & 0 & 0 & 0 & 0 & 0 & 0 \\
0 & 0 & 0 & 0 & 0 & 0 & 0 & 0 & 0 & 0 & 0 & 0 & 0 & 0 & 1 & 0 & 0 & -1 & -1 & 0 & 0 \\
0 & 0 & 0 & 0 & 0 & 0 & 0 & 0 & 0 & 0 & 0 & 0 & 0 & 0 & 0 & 0 & 0 & 0 & 1 & -1 & -1 \\
0 & 0 & 0 & 0 & 0 & 0 & 0 & 0 & 0 & 0 & 0 & 0 & 0 & 0 & 0 & 0 & 0 & 1 & 0 & 0 & -1 \\
0 & 0 & 0 & 0 & 0 & 0 & 0 & 0 & 0 & 0 & -1 & 0 & 0 & 0 & 0 & 0 & 0 & 0 & 0 & 0 & 1 \\
0 & 0 & 0 & 0 & 0 & 0 & 0 & 0 & 0 & 0 & 1 & 0 & 0 & 0 & 0 & 0 & 0 & 0 & 0 & -1 & 0
\end{bmatrix}
$$

3. Deriving fluxes from metabolomics data.

The dynamics of some fluxes can be directly derived from the metabolomics data with a stoichiometric model and the method of dynamic flux estimation (DFE); in other words, they do not require the use of a kinetic model.

Kinetic formulations for individual biochemical reactions. The following kinetic models surveyed from the literature were used in this study to compute fluxes under normal physiological conditions at discrete time points. To determine the enzymatic activity of the HK reaction, we used a kinetic formulation from the literature [2], namely:

$$Vglc\_g6p\_hk = (\frac{6300 \times MgATP}{Kmg\_ATP} + \frac{15435 \times MgATP \times Mg}{Kmg\_ATP \times Kmg\_ATP\_mg}) \times \frac{1}{D},$$

where

$$D = 1 + \frac{MgATP}{Kmg\_ATP} + \frac{Mg}{Kmg} + \frac{MgATP \times Mg}{Kmg\_ATP \times Kmg\_ATP\_mg} + (\frac{G6P}{Kglc\_g6p} + \frac{G16BP}{Kglc\_g16bp}) \times (1 + \frac{Mg}{Kmg}) + \frac{23BPG}{K23bpg} + \frac{Mg \times 23BPG}{Kmg \times Kmg\_23bpg}$$

Here, *Kmg_ATP* = 1440 µM, *Kmg_ATP_mg* = 1140 µM, *Kmg* = 1030 µM, *Kglc_g6p* = 69 µM, *Kglc_g16bp* = 69 µM, *K23bpg* = 2700 µM, and *Kmg_23bpg* = 3440 µM.

For the enzymatic activity of the PFK reaction, we used a kinetic formulation proposed by [2], namely:

$$Vf6p\_f16bp\_pfk = \frac{(\frac{K_{catf} \times MgATP \times F6P}{K\_MgATP \times K\_F6P} - \frac{K_{catr} \times F16BP \times MgADP}{K\_F16BP \times K\_MgADP})}{1 + \frac{MgATP}{K\_MgATP} + \frac{F6P}{K\_F6P} + \frac{MgATP \times F6P}{K\_MgATP \times K\_F6P} + \frac{F16BP}{K\_F16BP} + \frac{MgADP}{K\_MgADP} + \frac{F16BP \times MgADP}{K\_F16BP \times K\_MgADP}} \times \frac{1}{1+L}$$

, where

$$L = \frac{(\frac{[H^+]}{K_a})^4 (1 + \frac{ATP}{K\_ATP})^4 \times (1 + \frac{Mg}{K\_Mg})^4 (1 + \frac{23BPG}{K\_23BPG})^4}{(1 + \frac{F6P}{K\_F6P} + \frac{F16BP}{K\_F16BP})^4 \times (1 + \frac{AMP}{K\_AMP})^4 \times (1 + \frac{P_i}{K\_P_i})^4 \times (1 + \frac{G16BP}{K\_G16BP})^4}$$

Here *Kcatf* = 325512 µM/h, *Kcatr* = 14256 µM/h, *K_MgATP* = 68 µM, *K_F6P* = 75 µM, *K_F16BP* = 420 µM, *K_MgADP* = 540 µM, K_a = 8.9125E-8, *K_ATP* = 9.8 µM, *K_Mg* = 440 µM, *K_23BPG* = 1440 µM, *K_AMP* = 35 µM, K_P_i = 431 µM, and *K_G16BP* = 15.1 µM.

An alternative kinetic formulation for the same reaction was suggested by [2]:

$$Vf6p\_f16bp\_pfk = \frac{\text{Vmax\_pfk} \times \frac{F6P}{K\_F6P + F6P} \times \frac{MgATP}{K\_MgATP + MgATP}}{1 + L}.$$

where

$$L = L_0\_PFK \times \frac{(1.0 + \frac{MgATP}{K_{MgATP}})^4 \times (1.0 + \frac{Mg}{K\_Mg})^4}{(1.0 + \frac{F6P}{K\_F6P})^4 \times (1.0 + \frac{AMP}{K\_AMP})^4}.$$

Here, *Vmax_pfk* = *250000* µM/h, *K_F6P* = 74.4 µM, *K_MgATP* = 67.9 M, L_0*_PFK* = 0.0013 µM, K_{MgATP} = 83.4 µM, *K_Mg* = 443 µM, and *K_AMP* = 35 µM.

For the enzymatic activity of the ALD reaction, we used a kinetic model proposed by [3]

$$Vf16bp\_ga3p\_ald = \frac{(\frac{K_{catf}\times A}{K\_A} - \frac{K_{catr}\times P\times Q}{K\_P\times K_i\_Q})}{1+\frac{23BPG}{K_i\_23BPG}+\frac{A}{K\_A}+\frac{K\_Q\times P}{K\_P\times K_i\_Q}\times(1+\frac{23BPG}{K_i\_23BPG})+\frac{Q}{K_i\_Q}+\frac{K\_Q\times A\times P}{K_i\_A\times K\_P\times K_i\_Q}+\frac{P\times Q}{K\_P\times K_i\_Q}}$$

Here, *Kcatf* = 90576 µM/h, *Kcatr* = 311688 µM/h, *A* = F16BP, *K_A* = 7.1 µM, K_i_A = 19.8 µM, *P* = GA3P, *K_P* = 190 µM, *Q* = DHAP, *K_Q* = 35 µM, K_i_Q = 11 µM, and K_i_23BPG = 1500 µM.

For the enzymatic activity of the TPI reaction, we used a kinetic model by [4]

$$Vdhap\_ga3p\_tpi = \frac{(\frac{K_{catf}\times DHAP}{K\_DHAP} - \frac{K_{catr}\times GA3P}{K\_GA3P})}{1+\frac{DHAP}{K\_DHAP}+\frac{GA3P}{K\_GA3P}}$$

Here, *Kcatf* = 59754240 µM/h, *Kcatr* = 5253120 µM/h, *K_DHAP* = 162.4 µM, and *K_GA3P* = 446 µM.

For the rate of the PK reaction, we used the following kinetic formulation [5]:

$$Vpep\_pyr\_pk = \frac{(\frac{K_{catf}\times MgATP\times PEP}{K\_MgATP\times K\_PEP} - \frac{K_{catr}\times PYR\times MgADP}{K\_PYR\times K\_MgADP})}{1+\frac{MgATP}{K\_MgATP}+\frac{PEP}{K\_PEP}+\frac{MgATP\times PEP}{K\_MgATP\times K\_PEP}+\frac{PYR}{K\_PYR}+\frac{MgADP}{K\_MgADP}+\frac{PYR\times MgADP}{K\_PYR\times K\_MgADP}}\times\frac{1}{1+L}$$

where

$$L = \frac{(\frac{[H^+]}{K_a})(1+\frac{ATP}{K\_ATP})^4}{(1+\frac{PEP}{K\_PEP}+\frac{PYR}{K\_PYR})^4\times(1+\frac{F16BP}{K\_F16BP}+\frac{G16BP}{K\_G16BP})^4}$$

Here, *Kcatf* = 434095 µM/h, *Kcatr* = 1021 µM/h, *K_MgATP* = 3000 µM, *K_PEP* = 225 µM, *K_PYR* = 2000 µM, *K_MgADP* = 474 µM, $K_a = 10^{-6.8}$, *K_ATP* = 3390 µM, *K_F16BP* = 5 µM, and *K_G16BP* = 100 µM.

Table S1. Demographics of the donors.

Donor ID	Gender	Race	Age
X850	Female	Black	38
X867	Female	White	42
X1145	Male	White	51
X1	Male	White	55
X2	Male	Black	60
X3	Male	Black	46
X4	Male	White	55
X5	Male	Black	64
X6	Male	Black	47

Table S2. Influence of secondary factors on storage time effects.

Influential factors	Influence on storage time effects	Example
**Donation batch**	Differences among batches increase during the first two weeks of storage, and then decrease until the end of storage. Donor variations are much smaller than batch variations, especially during weeks 2 to 4 of storage.	HK
**Reaction dependency**	Storage time effects are reaction dependent, and this reaction dependency is consistent among donors.	ALD, HK, and PK
**Kinetic model**	Quantification of storage effects on a specific flux is consistent for all donors in regard to different kinetic models.	PFK

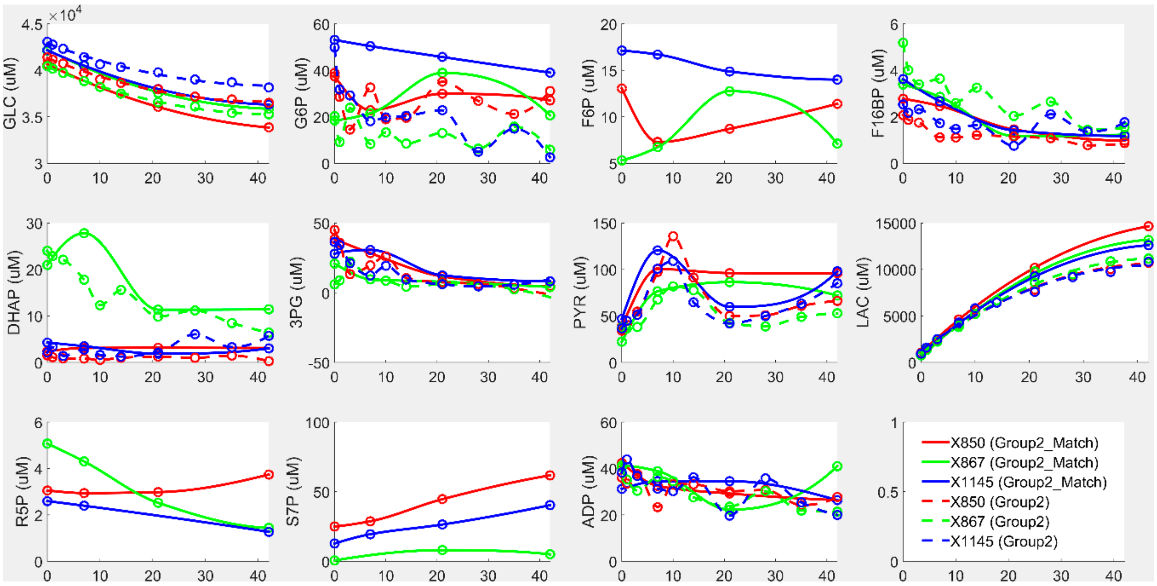

Figure S1. Dynamics of metabolite levels for donors in group2 and group2_match. After conversion to absolute concentrations, the data were calibrated according to the signal intensity of carbon atoms in consideration of 5–6% instrument variability and 13–18% total process variability. Shown here are the dynamic levels of metabolites for donors in group2_match and group2. Symbols are experimental data points, while lines represent the interpolated data set. The X-axis shows storage time (unit: days), while the Y-axis represents absolute metabolite concentrations (unit: μM).

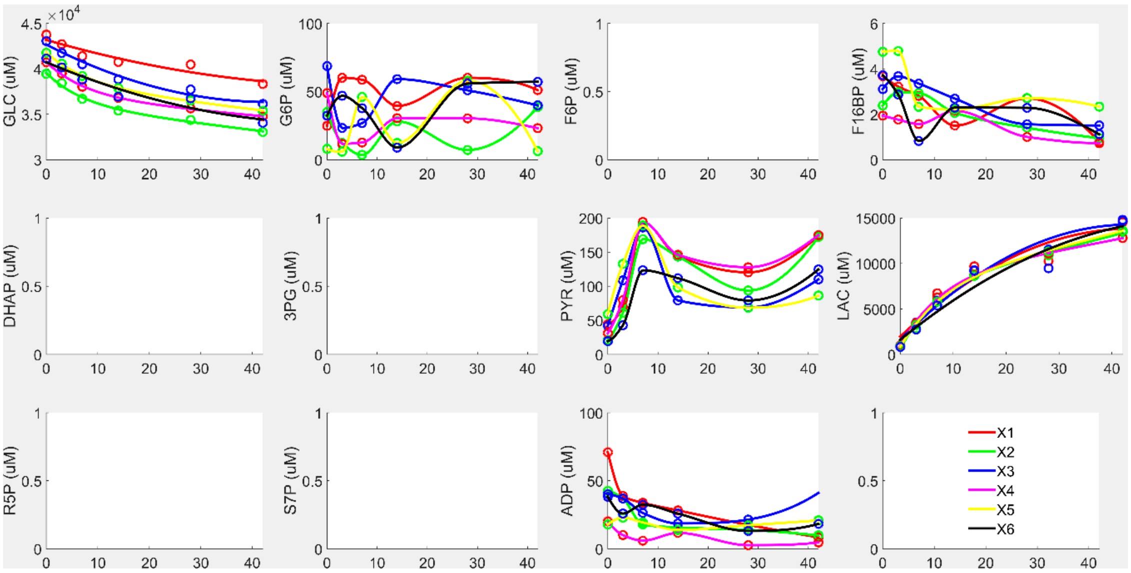

Figure S2. Dynamics of metabolite levels for donors in group1. After conversion to absolute concentrations, the data were calibrated according to the signal intensity of carbon atoms in consideration of 5–6% instrument variability and 13–18% total process variability. Shown here are dynamic levels of metabolites for the donors in group1. Symbols are experimental data points, while lines represent the interpolated data set. The X-axis shows storage time (unit: days), while the Y-axis represents absolute metabolite concentrations (unit: μM). For easier comparison with Figure S1, the plots are arranged in the same manner. Some metabolite measurements were not available.

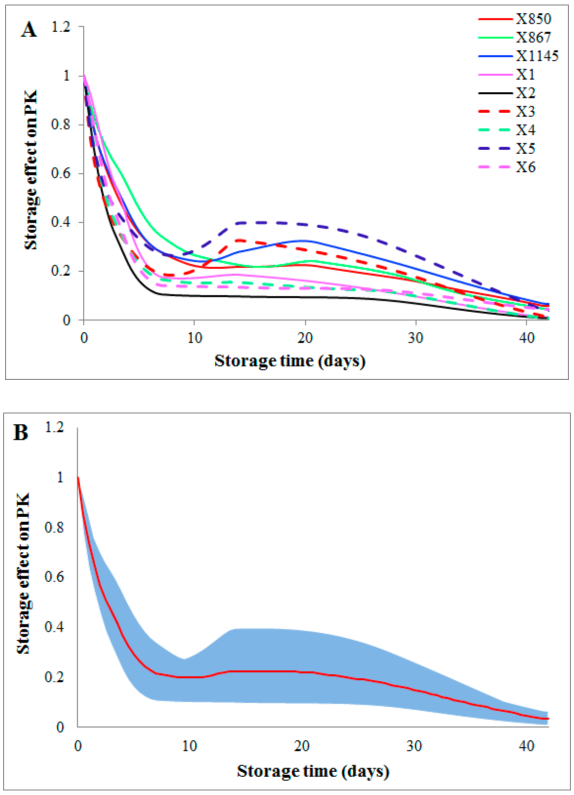

Figure S3. Storage effect on the PK flux among all donors. Storage time effects on the PK flux are quantified and compared among all donors. Coloring schemes and line styles are indicated in the inset legend. The X-axis represents storage time (unit: days), while the Y-axis shows the effect on the activity of enzyme PK. A: Two batches of donations from the same donors (group2 and group2_match) are averaged; B: All donations are averaged (the red curve) and the dynamic ranges of variations among donors during storage are shown as the blue area.

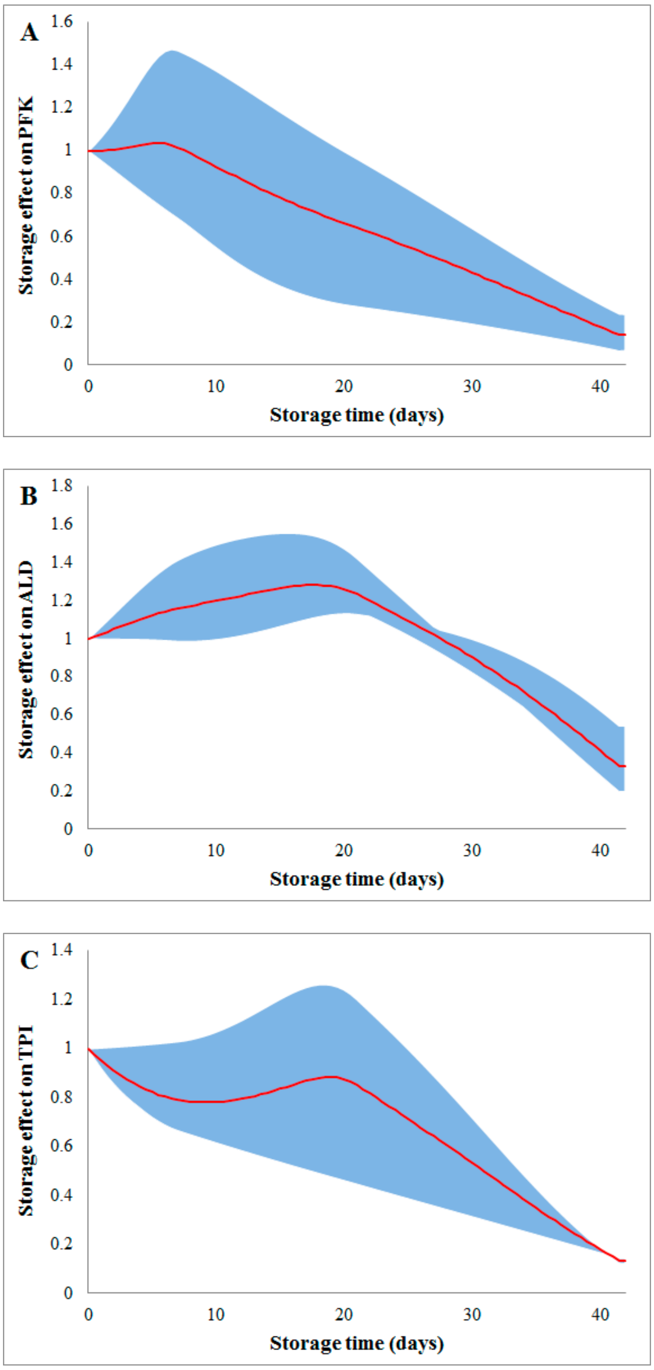

Figure S4. Storage effect on fluxes PFK, ALD, and TPI. Storage time effects on fluxes PFK, ALD, and TPI are quantified and compared among all donors. Donors are averaged (the red curve) and the dynamic ranges of variations among donors during storage are shown as the blue areas. The X-axis represents storage time (unit: days), while the Y-axis shows the effect on the activity of corresponding enzymes. A: flux PFK; B: flux ALD; C: flux TPI.

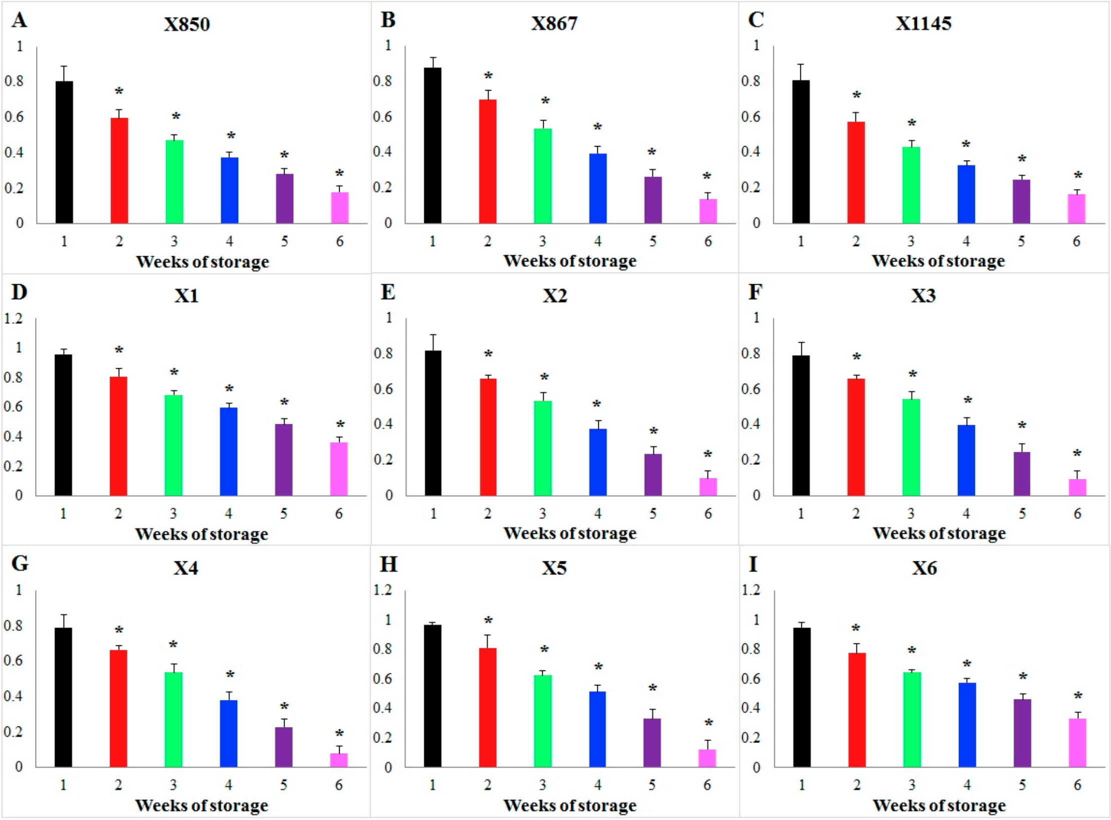

Figure S5. Comparison of storage time effect on the HK flux during different weeks of storage for each donor. Storage time effects on the HK flux are compared week by week for each donor. Bar graphs show the weekly averaged storage effects and standard deviations. The X-axis represents storage time (unit: weeks), while the Y-axis shows the effect on the activity of enzyme HK. Each subplot represents the comparison in a donor whose ID is shown in its title, A: donor X850; B: donor X867; C: donor X1145; D: donor X1; E: donor X2; F: donor X3; G: donor X4; H: donor X5; I: donor X6. Significance level: * ($P < 0.01$) for the statistical difference between a corresponding week (2–6) and 1st week in terms of weekly averaged storage time effects.

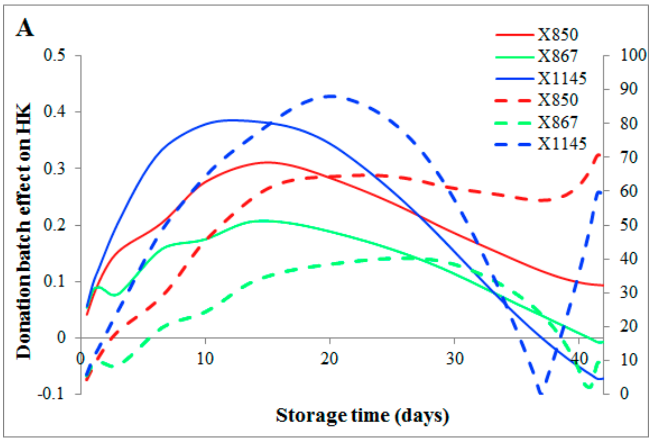

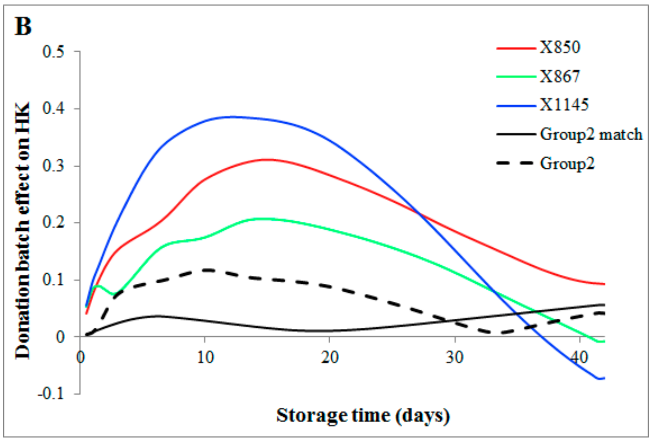

Figure S6. Influence of donation batches on storage time effect on the flux HK. Influences of donation batches are quantified for the two batches of blood donations and the difference in storage time effects on the flux HK is shown for each donor. The X-axis represents storage time (unit: days). A: The Y-axis on the left shows absolute donation batch differences (solid lines), while the Y-axis on the right shows relative magnitudes of donation differences in comparison to the average storage time effect (dashed lines). B: The Y-axis shows absolute donation batch differences (solid color lines), donor variation in Group2_Match (solid black line), and donor variation in Group2 (dashed black line).

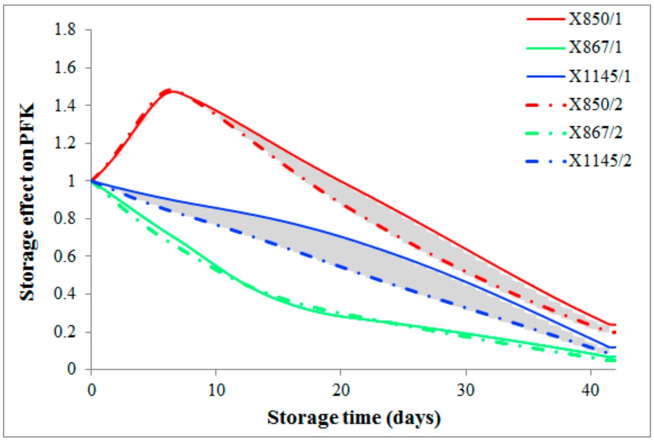

Figure S7. Influence of different kinetic models. Two available kinetic models for the flux PFK were used to quantify storage time effect. Solid lines represent results from one model, while dashed lines are from the other model. Grey areas show the differences in storage time effect between two models for individual donors. The X-axis represents storage time (unit: days), while the Y-axis shows storage time effect.

References

1. Roback, J.D.; Josephson, C.D.; Waller, E.K.; Newman, J.L.; Karatela, S.; Uppal, K.; Jones, D.P.; Zimring, J.C.; Dumont, L.J. Metabolomics of adsol (as-1) red blood cell storage. *Transfusion medicine reviews* **2014**, *28*, 41-55.
2. Levit K, R.K., Elixhauser A, Stranges E, Kassed C, Coffey R. Hcup facts and figures: Statistics on hospital-based care in the united states in 2005.
3. Dumont, L.J.; AuBuchon, J.P. Evaluation of proposed fda criteria for the evaluation of radiolabeled red cell recovery trials. *Transfusion* **2008**, *48*, 1053-1060.
4. van 't Erve, T.J.; Wagner, B.A.; Martin, S.M.; Knudson, C.M.; Blendowski, R.; Keaton, M.; Holt, T.; Hess, J.R.; Buettner, G.R.; Ryckman, K.K., *et al.* The heritability of metabolite concentrations in stored human red blood cells. *Transfusion* **2014**, *54*, 2055-2063.
5. Van 't Erve, T.J.; Wagner, B.A.; Martin, S.M.; Knudson, C.M.; Blendowski, R.; Keaton, M.; Holt, T.; Hess, J.R.; Buettner, G.R.; Ryckman, K.K., *et al.* The heritability of hemolysis in stored human red blood cells. *Transfusion* **2015**, *55*, 1178-1185.
